# Frequency of β-thalassemia trait and other hemoglobinopathies in northern and western India

**DOI:** 10.4103/0971-6866.64941

**Published:** 2010

**Authors:** Nishi Madan, Satendra Sharma, S. K. Sood, Roshan Colah, (Late) H. M. Bhatia

**Affiliations:** Department of Pathology, University College of Medical Sciences and Guru Teg Bahadur Hospital, Delhi 110 095, India; 1 National Institute of Immunohaematology (Indian Council of Medical Research), KEM Hospital, Parel, Mumbai, India

**Keywords:** β-thalassemia trait, Hemoglobinopathies, India

## Abstract

**INTRODUCTION:**

India is an ethnically diverse country with an approximate population of 1.2 billion. The frequency of beta-thalassemia trait (βTT) has variously been reported from <1% to 17% and an average of 3.3%. Most of these studies have been carried out on small population groups and some have been based on hospital-based patients. There is also a variation in the prevalence of hemoglobinopathies in different regions and population groups in the country. A high frequency of Hb D has been reported from the North in the Punjabi population, Hb E in the eastern region of India and Hb S is mainly reported from populations of tribal origin from different parts of the country.

**OBJECTIVES:**

To study the gene frequency of βTT and other hemoglobinopathies in three regions East (Kolkata), West (Mumbai) and North (Delhi) in larghe population group (schoolchildren) for a more accurate assessment of gene frequency for planning of control programmes for haemoglobinopathies.

**MATERIALS AND METHODS:**

This study included 5408 children from 11 schools in Delhi, 5682 from 75 schools in Mumbai and 957 schoolchildren from Kolkata who were screened for βTT and haemoglobinopathies. These included 5684 children from 75 schools in Mumbai and 5408 children from 11 schools in Delhi. Children were 11-18 years of age of both sexes. The final report is, however, only on 11090 schoolchildren from Mumbai and Delhi as data from Kolkata was restricted both in numbers and objectives and could not be included for comparison.

**RESULTS:**

The overall gene frequency of βTT in Mumbai and Delhi was 4.05% being 2.68% and 5.47% in children of the two cities respectively. In Mumbai, the gene frequency was evenly distributed. Majority of the children with βTT from Mumbai were from Marathi (38.9%) and Gujarati (25%) speaking groups. Gene frequency was >5% in Bhatias, Khatris, Lohanas and Schedule Castes. In Delhi, a higher incidence was observed in schoolchildren of North and West Delhi (5.8-9.2%). The schoolchildren of North and West Delhi comprised predominantly of Punjabi origin compared to children in the South of the city (2.2%, 2.3%). When analyzed state-wise, the highest incidence was observed in children of Punjabi origin (7.6%) and was >4% from several other states. Majority of the traits from Mumbai were anemic (95.1% male and 85.6% in female). The prevalence of anemia was lower (62.7% male and 58.4% female) children with βTT from Delhi. This was a reflection of the higher prevalence of anemia in children without hemoglobinopathy in Mumbai than in Delhi. Nutritional deficiency was probably more severe and rampant in children Mumbai. Gene frequency of Hb D was greater in schoolchildren from Delhi (1.1%) than in Mumbai (0.7%). Hb S trait (0.2%) was observed exclusively in children from Mumbai. A low incidence of Hb E trait (0.04%) was seen in children in Mumbai. A higher incidence is reported from the East. The number of cases studied from the eastern region was small as the data from the East (Kolkata) could not be included in the analysis.

**CONCLUSION:**

This study comprises a larger number of children studied for the gene frequency of βTT and other hemoglobinopathies from India. Population groups with higher gene frequencies require screening programmes and facilities for antenatal diagnosis as well as increased awareness and educational programmes to control the birth of thalassemic homozygotes. The overall carrier frequency of βTT was 4.05% and reinforces the differential frequency of β-thalassemia trait in schoolchildren from Delhi and Mumbai and the higher incidence of hemoglobin D in Punjabis as reported previously. The birth incidence calculated thereof for homozygous thalassemics would be 11,316 per year which are added each year to the existing load of homozygous thalassemics. This is much higher than the previously reported number of births annually. Hence suitable control measures need to be undertaken urgently in India.

## Introduction

Birth rates of homozygous β-thalassemia in different parts of the world have reduced considerably. Some smaller countries have reported no newborns with the disease. This has been achieved by control programs involving screening population surveys for heterozygous β-thalassemia, antenatal diagnosis along with increasing awareness in the medical profession, and in the population by large-scale education and counseling. Control programs in Sardinia have substantially reduced the birth of homozygous thalassemics from 1:250 to as low as 1:4000 births.[1]

Estimates of newborns with homozygous β-thalassemia in India vary considerably from 6,000 to 7,500 per year and even more depending on the gene prevalence, population, and birth rate of the region.[2] These are added annually to the already existing homozygote population. A frequency of β-thalassemia trait (βTT) of 1–3% and an overall 3.3% is stated for India.[3] Based on this figure, an estimate of 7,500 expected homozygous births per year have been made.[4] There is, therefore, a considerable discrepancy between the two estimates. So what is the true gene frequency? There are several reports of the incidence of βTT from different parts of the country, which vary from less than 1% to 17%.[5,6] Most of the earlier studies are in small groups of hospital-based patients and/or population groups. It is also known that the incidence is higher in some population groups.[7] There is a stated lack of information about the true gene frequency in many parts of the world.[8] Prevalence data preferably require large-scale population surveys and should not be hospital-based.[8] It is essential to have a more accurate assessment of the gene frequency of βTT in the population for planning control programs for β-thalassemia in the country.

India is a vast country with considerable regional and ethnic heterogeneity. A study of every region is an impossible proposition due to lack of infrastructural facilities, expertise, and resources.

In view of this, the Indian Council of Medical Research Advisory Committee on Hematology recommended the formation of a Task Force to obtain information on the prevalence of β-thalassemia and other hemoglobinopathies in different regions in the country (ICMR).

It was, therefore, decided to focus attention on three cities, with a large population and well-established laboratory facilities. Mumbai, Delhi, and Kolkata were selected because they represent three different zones of the country. The population studied included school children from these cities. At the same time, such a study design would generate data on regional and ethnic variation, if any.

The results obtained in 11,090 school children from two of the three regions are based on the findings of the ICMR Collaborative Study Report.[9] This is the largest published study to date on the frequency of βTT and other hemoglobinopathies in India to the best of our knowledge.

## Materials and Methods

The three institutions collaborating in this multicentric study were:

1. University College of Medical Sciences, Delhi.
2. Institute of Immunohaematology (IIH) now National Institute of Immunohaematology (NIIH), Indian Council of Medical Research, Mumbai.
3. School of Tropical Medicine, Kolkata.

This study included 5408 children from 11 schools in Delhi, 5682 from 75 schools in Mumbai, and 957 school children from Kolkata. The study in Kolkata was restricted both in numbers and objectives, and the data could not be included for comparison. The study was restricted to secondary school children in the age group of 11 to 18 years. Schools were more or less randomly selected, but an attempt was made to include most of the local population from different areas in the cities. This included children from all sections of society from different castes and religious groups. After obtaining the necessary permission from the Education Departments, the respective school principals were approached. An informative write-up on thalassemia was sent to the parents and after getting their consent, blood collection was organized during school hours. Children were also clinically examined by the medical officers of the respective teams.

Two to three milliliter blood was collected in EDTA for complete blood count (CBC) and analysis of hemoglobin variants. Parents and siblings of children found to have any hemoglobinopathy were called for investigation for βTT and follow-up for counseling if required.

CBC was measured by the Erma PC 604 particle counter. Peripheral blood smears were examined after staining with Wright’s stain.[10] Hemoglobin electrophoresis[10] was carried out on cellulose acetate using TEB buffer, pH 8.6.[10] HbA_2_ was estimated following elution after electrophoresis on cellulose using acetate, TEB buffer, pH 8.9.[11] HbA_2_ was estimated in all the school children and the cut-off for HbA_2_ was >3.5%. Confirmation was carried out by investigating parents and/or siblings. Hemoglobin F was quantitated in children by Singer’s alkali denaturation method[12] in Mumbai and by Betke’s method[13] in Delhi. Solubility and sickling test were done in all cases with hemoglobin bands in the HbD/HbS region.[10]

## Results

**Figure 1:**
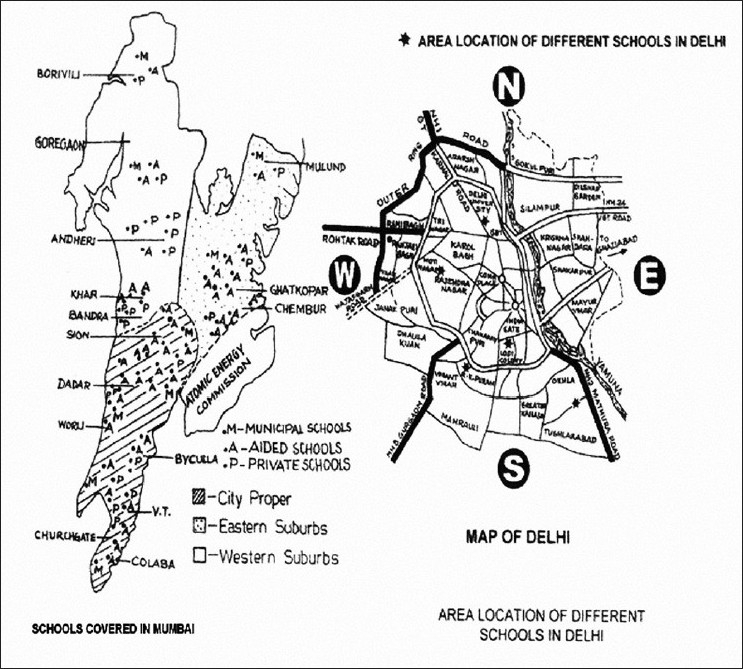
Location of schools in Mumbai and Delhi

Total 11,090 school children were screened for TT and hemoglobinopathies. These included 5684 children from 75 schools in Mumbai and 5408 children from 11 schools in Delhi. The location of the schools included in the study in Mumbai and Delhi are shown in Figure 1.

The male–female ratio of children investigated was 1:1 at Mumbai and 1.4:1 at Delhi.

### Prevalence of hemoglobinopathies

Table 1 shows the prevalence of βTT and Table 2 the prevalence of other hemoglobinopathies (e.g., HbD, HbS, HbE trait, and HPFH/δβ-thalassemia) in 5682 school children from Mumbai and 5408 from Delhi. HbS trait was present in 0.16% in children in Mumbai, HbD trait in 0.66% in Mumbai and 1.10% in Delhi, and HbE trait in 0.04% in Mumbai.

**Table 1:** Frequency of β-thalassemia trait in Mumbai and Delhi

City	No. Studied	No. with βTT	%
Mumbai	5682	152	2.68
Delhi	5408	296	5.47
Total	11,090	449	4.05

**Table 2:** Frequency of other Hb variants detected in school children in Mumbai and Delhi.

	Mumbai			Delhi			Total		
	No. Studied	No.	%	No. Studied	No.	%	No. Studied	No.	%
Hb-D Punjab	5682	38	0.7	5408	59	1.1	11090	97	0.9
Hb-S trait	5682	9	0.2	5408	0	0	11090	9	0.1
βδ -Thal/ HPFH	5682	12	0.2	5408	14	0.3	11090	26	0.2
HbE trait	5682	2	0.04	5408	0	0	11090	2	0.02

### Prevalence of β-thalassemia trait

The overall frequency of βTT in the school children was 4.05% being 2.68% and 5.49% in school children in Mumbai and Delhi, respectively [Table 1].

In Delhi, the prevalence was higher in children from schools in the North and West (5.8–9.2%) of the city as compared to school children from South Delhi (2.2%, 2.3%) [Table 3]. In school children from Delhi, the incidence of βTT ranged from 2.2% to 9.2%. Majority of the children in schools in the North and West originated from Punjab. In a school from Central Delhi, in which the population was predominantly of Karnataka origin, the frequency was 6.4%.

**Table 3:** Frequency of β-thalassemia trait in school children in Delhi

School	No. Screened	No.	%
South	838	19	2.3
South	1187	26	2.2
Central	420	27	6.4
West	842	49	5.8
West	433	39	9.0
West	563	47	8.3
North	424	39	9.2
North	701	50	7.1
Total	5408	296	5.47

Table 4 shows the frequency of βTT in the school children in Delhi analyzed according to their state of origin. Majority of the children screened were from Punjab (40.4%) and Uttar Pradesh (25.02%). The frequency varied from 2.4% to 7.6% in children belonging to different states, being the highest (7.6%) among the children from Punjab.

**Table 4:** Frequency of β-thalassaemia trait in school children from Delhi according to the state of origin

State or Origin	No. Screened	β-thalassemia Trait	
		No.	%
Punjab	2213	168	7.6
Uttar Pradesh	1353	56	4.1
Delhi	434	19	4.4
Haryana	397	14	3.5
Rajasthan	251	6	2.4
Himachal Pradesh	69	4	5.8
Kashmir	31	2	6.5
Karnatak	261	17	6.5
Tamil Nadu	39	2	5.1
Bihar	83	4	4.8
Maharashtra	35	1	2.9
Sindh	34	2	5.9
Othersa	208b	1b	0.5

In Mumbai, the frequency of βTT in different caste groups is shown in Table 5. Nineteen different caste groups showed the presence of βTT in a frequency ranging from 1.0% to 6.9%. It was >5% in Bhatias, Khatris, Lohanas, and scheduled caste. Majority of children with βTT from Mumbai were from Marathi (38.9%) and Gujarati (25.0%) speaking groups. In Delhi, accurate information regarding caste distribution was not possible to elicit. Therefore, an attempt to categorize children according to caste was made more often from family names. Majority of the children could not be categorized.

**Table 5:** Frequency of β-thalassemia trait in school children in Mumbai and Delhi according to caste/religion

Group	Mumbai		Delhi	
	No./Total	%	No./Total	%
Artisan	13/506	2.6	1/23	4.3
Baniya	4/133	3.0	10/369	2.7
Bhandari	0/145	0	0/12	0
Bhatia	4/68	5.9	1/32	3.1
Bhayya	1/66	1.5	0/18	0
Bohri	3/76	4.0	0/1	0
Brahmin	15/764	2.0	64/1201	5.3
Buddhist	8/222	3.6	1/8	1.3
Irani	0/1	0	0	0
Jain	16/488	3.3	4/83	4.8
Jath	0/20	0	4/58	6.9
Kayastha	3/88	3.4	5/94	5.3
Khatri	5/73	6.9	19/496	3.8
Kunbi	2/91	2.2	0/4	0
Lohana	9/162	5.6	0/12	0
Maratha	23/1100	2.1	0	0
Parsee	0/45	0	0/2	0
Protestant	2/49	4.1	0/3	0
Roman Catholic	2/207	1.0	2/34	5.9
Scheduled Caste	11/214	5.1	7/290	2.4
Shiya	5/180	2.8	0/4	0
Sikh	4/108	3.7	12/260	4.6
Sunni	7/282	2.5	1/60	1.7
Others/ Not known	15/594	2.5	130/2344	5.5
Total	5682		5408	

### Prevalence of anemia in children with β-thalassemia trait

Table 6 shows the distribution of Hb concentration in βTT. Hb concentration of <9 g/dl was present in 9.2% of female children in Mumbai and 1.2% of male and 3.2% of female children in Delhi. Anemia (WHO criteria) was present in 96.1% male and 85.6% female children in Mumbai and 62.7% male and 58.4% female children in Delhi. Hb concentration of >14 g/dl was present in 18.7% of male and 3.2% of female children in Delhi. In Mumbai, there were no heterozygous β-thalassemics with Hb >14/dl. Table 7 shows the hematologic parameters in children with βTT.

**Table 6:** Distribution of Hb concentration in all cases of β-thalassemia trait

Hb (g/dl)	Mumbai						Delhi					
	Male			Female			Male			Female		
	No.	%	Cum%	No.	%	Cum%	No.	%	Cum%	No.	%	Cum%
<8.0	0	0	0	3	3.9	3.9	1	0.6	0.6	3	2.4	2.4
8.0–8.9	0	0	0	4	5.3	9.2	1	0.6	1.2	1	0.8	3.2
9.0–9.9	11	14.5	14.5	11	14.5	23.7	7	4.1	5.3	7	5.6	8.8
10.0–10.9	23	30.3	44.8	31	40.8	64.5	21	12.3	17.6	20	16.0	24.8
11.0–11.9	22	28.9	73.7	16	21.1	85.6	29	16.9	34.6	42	33.6	58.4
12.0–12.9	17	22.4	96.1	9	11.0	97.4	48	28.1	62.7	29	23.2	81.6
13.0–13.9	03	3.9	100	2	2.6	100	32	18.7	81.3	19	15.2	96.8
>14.0	0	0	0	0	0	0	32	18.7	95.3	4	3.2	100
Total	76			76			171			125		

**Table 7:** Hematological parameters in all cases of β-thalassemia trait.

Investigations	Mumbai			Delhi		
	Male	Female	Total	Male	Female	Total
Hb (g/dl)	11.2 ± 1.1	10.6 ± 1.2	10.9 ± 1.1	12.5 ± 1.6	11.7 ± 1.4	12.2 ± 1.6
Hct (%)	35 ± 3	34 ± 4	35 ± 4	38 ± 5	37 ± 4	38 ± 4
RBC (×1012/l)	5.7 ± 0.6	5.3 ± 0.5	5.5 ± 0.6	5.3 ± 0.7	5.1 ± 0.6	5.25 ± 0.7
MCV(fl)	62 ± 6	63 ± 7	62 ± 7	74 ± 11	72 ± 10	73 ± 10
MCH (pg)	19.6 ± 1.9	20.1 ± 2.3	19.8 ± 2.1	24.1 ± 4	23.2 ± 3.5	23.7 ± 3.8
MCHC (g/dl)	31.5 ± 1.9	31.9 ± 2.9	31.7 ± 2.5	32.6 ± 2.1	32.1 ± 1.6	32.4 ± 1.9
HbA2 (%)	5.38 ± 0.69	5.04 ± 0.67	5.21 ± 0.68	4.6 ± 0.9	4.6 ± 0.9	4.6 ± 0.9
HbF (%)	0.72 ± 0.5	0.71 ± 0.4	0.71 ± 0.4	1.5 ± 0.9	1.9 ± 0.9	1.6 ± 0.9

Values are expressed as mean±SD.

### Prevalence of anemia in children without any hemoglobinopathy

Table 8 shows the prevalence of anemia in all school children without any hemoglobinopathy. In Mumbai, 28.4% of male and 30.5% of female children were anemic. In Delhi, 13.7% of male and 18.6% of female children were anemic. The overall prevalence of anemia was 29.6% in children in Mumbai and 15.8% in Delhi.

**Table 8:** Prevalence of anemia in all school children without any hemoglobinopathy

Sex	Mumbai			Delhi			All cases		
	Total	Anemic		Total	Anemic		Total	Anemic	
	No.	No.	%	No.	No.	%	No.	No.	%
Male	2718	771	28.4	3014	413	13.7	5732	1184	20.7
Female	2754	840	30.5	2298	427	18.6	5052	1267	25.1
Total	5472	1611	29.6	5312	840	15.8	10784	2451	22.7

### Prevalence of hepatosplenomegaly in children with β-thalassemia trait

In Mumbai, 19.7% children with βTT had hepatomegaly and 2.0% had splenomegaly; only a few children (0.7%) in Delhi had an enlarged liver and spleen.

### HbA_2_ concentration

HbA_2_ levels ranged from 3.8% to 7.5% and 3.5% to 7.5% in children from Mumbai and Delhi, respectively [Table 9].

**Table 9:** HbA_2_ levels in school children with β-thalassemia trait

HbA_2_ (%)	Mumbai			Delhi		
	No.	%	Cum%	No.	%	Cum%
3.5–4.0	6	4.0	4.0	106	35.8	35.8
4.1–4.5	26	17.1	21.1	72	24.3	60.1
4.6–5.0	30	19.7	40.8	41	13.9	74.0
5.1–5.5	42	27.6	68.4	26	8.8	82.8
5.6–6.0	28	18.4	86.8	25	8.4	91.2
6.1–6.5	16	10.5	97.3	13	4.4	95.6
6.6–7.0	3	2.0	99.3	7	2.4	98.0
7.1–7.5	1	0.7	100.0	6	2.0	100
Total	152			296		

### HbF concentration

Distribution of the HbF concentration in 152 school children with βTT from Mumbai and 284 from Delhi is shown in Table 10. In Mumbai, HbF concentration varied from 0% to 2% and in Delhi from 0% to >5%.

**Table 10:** Distribution of HbF in school children with β-thalassemia trait

HbF (%)	Mumbai			Delhi			Total		
	No.	%	Cum%	No.	%	Cum%	No.	%	Cum%
<1.0	122	80.3	80.3	88	31.0	31.0	210	48.2	48.2
1.1–1.5	21	13.8	95.1	70	24.6	55.6	91	20.9	69.1
2.1–2.5	9	5.9	100	40	14.1	69.7	49	11.2	80.3
2.1–2.5	0	0		38	13.4	83.1	38	8.7	89.0
2.6–3.0	0	0		29	10.2	93.3	29	6.6	95.6
3.1–3.5	0	0		12	4.2	97.5	12	2.8	98.4
3.6–4.0	0	0		0	0.0	97.5	0	0.0	98.4
4.1–4.5	0	0		5	1.7	99.2	5	1.2	99.6
4.6–5.0	0	0		1	0.4	99.6	1	0.2	99.8
>5.0	0	0		1	0.4	100.0	1	0.2	100.0

### Prevalence of high HbF

The hematological findings in school children with high HbF in Mumbai and Delhi are shown in Tables 11 and 12. Twelve children in Mumbai (0.2%) and 14 in Delhi (0.3%) had a raised HbF (>5%) with a normal HbA_2_. These cases could either be heterozygotes for δβ-thalassemia or hereditary persistence of fetal hemoglobin (HPFH). The HbF levels in these cases in Mumbai varied from 5.5% to 23.0%, while in Delhi they ranged from 5.3% to 10.6%. All of them had an Hb of >10 g/dl except one case in Mumbai who had an Hb concentration of 8.4 g/dl. Seven of the 12 cases from Mumbai had an MCV of <75 fl. However, in Delhi, the MCV was <75 fl in only 2 of the 14 cases while 3 cases had increased MCV levels (>100 fl). Further investigations are required to differentiate children with dβ-thalassemia trait from those having HPFH trait.

**Table 11:** Hematological findings in school children with high HbF from Mumbai.

Case	Sex	Hb (g/dl)	Hct (%)	RBC (×10^12^/l)	MCV (fl)	MCH (pg)	MCHC (g/dl)	HbF (%)	HbA_2_ (%)	G6PD
1	M	11.9	38	5.47	70	21.7	30.6	6.5	0.8	N
2	M	13.0	45	6.30	72	20.5	28.3	5.5	3.1	N
3	M	12.5	36	5.39	67	23.1	34.4	6.2	1.6	N
4	F	08.4	31	4.4	71	19.1	6.4	7.3	2.0	N
5	F	12.8	40	4.90	81	25.9	31.9	12.4	—	N
6	M	15.1	46	5.7	81	26.1	32.2	23.0	—	N
7	M	14.3	49	5.7	86	25.0	29.0	16.8	—	N
8	F	12.2	35	4.6	75	26.4	34.8	19.1	—	N
9	M	12.4	39	4.9	79	25.4	31.9	12.2	—	N
10	F	14.9	39	5.3	74	28.1	37.5	22.4	1.8	N
11	F	13.7	37	5.1	72	6.5	36.6	20.0	—	N
12	F	12.0	39	5.6	69	21.5	31.3	18.4	2.2	N

**Table 12:** Hematological findings in school children with high HbF from Delhi

Case	Sex	Hb (g/dl)	Hct (%)	RBC (×1012/l)	MCV (fl)	MCH (pg)	MCHC (g/dl)	HbF (%)	HbA_2_ (%)	G6PD
1	M	12.8	40	5.77	69	22.2	30.5	7.8	2.2	N
2	F	11.6	34	3.24	105	35.8	34.1	8.7	0.0	N
3	M	10.7	32	3.16	102	33.8	33.4	5.2	1.3	N
4	F	13.5	42	5.45	77	24.7	32.1	9.4	3.4	N
5	M	12.5	38	4.49	84	27.8	32.9	6.6	2.0	N
6	M	15.2	47	5.24	88	29.0	32.3	8.5	3.1	N
7	F	13.0	40	3.73	106	34.8	32.5	10.6	3.3	N
8	M	11.7	35	4.17	83	28.0	33.4	3.5	1.9	N
9	M	13.7	41	5.08	80	26.9	33.4	5.1	2.0	N
10	M	12.4	37	4.66	79	26.6	33.5	9.4	2.6	N
11	M	12.5	38	5.08	74	24.6	32.9	7.1	3.0	N
12	M	12.1	35	4.68	75	25.8	34.6	5.4	1.6	N
13	F	13.6	41	4.95	81	27.4	33.2	5.3	1.7	N
14	M	15.4	45	5.64	80	27.3	34.2	6.4	1.2	N

## Discussion

The primary objective of this large multicentric study was to determine the frequency of β-thalassemia and other hemoglobinopathies in different regions in the country. The study was restricted to secondary school children as they were considered to be fairly representative of all sections of the population. Besides they also comprised a population group which could be easily accessible for investigations and counseling.

Significant regional differences were observed in the prevalence of anemia both in children with βTT and in those without any Hb variant. Prevalence of anemia was lesser in the North as compared to the Western part of the country [Table 8].

In 1975, Sukumaran had observed that β-thalassemia is probably the commonest inherited hemoglobin disorder on the Indian subcontinent.[6] HbE was more frequent in the east. The collaborative study on thalassemia in the two regions carried out on 11,090 school children establishes that βTT is the most frequent hemoglobinopathy of clinical importance although it varies considerably in the two regions and in different communities/caste groups and regions. The incidence was higher in the North (Delhi) being 5.5% than in the West (Mumbai) being 2.7%. The overall frequency was 4.05%. The lower incidence in Mumbai is probably due to the greater heterogeneity of the population screened.

The frequency of βTT varied widely in Delhi varying from 2.2% to 9.2%. It was interesting to note the higher frequency of βTT in children from schools in the West and North of Delhi (5.8–9.2%) where the population is largely of Punjabi origin compared to children from the south (2.2%, 2.3%) of the city [Table 3]. One school in Central Delhi had a predominant population of school children of Karnataka origin in whom the incidence was 6.4%. The population of Delhi is largely of Punjabi origin since the partition of India when large segments of population from Western Punjab (now Pakistan) migrated to Punjab, Delhi, and surrounding areas. Although the number of school children from Himachal Pradesh, Kashmir, Karnataka, Tamil Nadu, Bihar, and Sindh were small, the frequency was significant ranging from 4.8% to 6.5%. The lowest prevalence of βTT was observed in school children from Rajasthan and Maharashtra. In Delhi, there was less heterogeneity in the population, although number of children in some groups was restricted for a meaningful comparison.

Unlike the prevalence rate which varied considerably in school children from Delhi, in Mumbai it was evenly distributed in the city proper (2.8%), Western suburbs (2.34%), and the Eastern suburbs (2.72%). There was no significant difference in the prevalence of βTT in different schools as majority of them catered to a similar although cosmopolitan and heterogeneous population. Nevertheless, it was noted that hardly any caste, or religious group, or population group from any state was free from β-thalassemia. It is interesting to note, however, that of 35 Maharashtrians screened in Delhi, 1 (2.9%) was heterozygous for β-thalassemia.

In Mumbai, the prevalence of β-thalassemia carriers varied from 0% to 6.9% in different caste and/or religious groups. It was greater than 4% in the Bhatia (5.9%), Bohri (4%), Khatri (6.9%), Lohana (5.6%), Protestants (4.1%), and scheduled castes (5.1%) [Table 5]. Although a similar attempt at grouping was made in Delhi, most school children and parents were unable to provide details regarding their caste group. In Delhi, the frequency varied from 0% to 6.9% in these groups. The largest group of 2146 classified as “others” because of unavailability of detailed caste had a frequency of 6.05% and belonged largely to migrant Punjabi population. The lower incidence in some groups known to have higher incidence of βTT is likely to be due to the smaller numbers of school children screened in each group.

In the neighboring country of Pakistan, the frequency of β-thalassemia has been reported to be 5.6%,[14] which is similar to that observed in school children from Delhi with a largely Punjabi population.

The prevalence of anemia was higher in children with βTT from Mumbai compared to Delhi. A higher number of children from Delhi were not anemic (39.2%) compared to Mumbai (8.9%), possibly indicating a higher prevalence of iron deficiency in the latter.

HbS trait was seen in children in Mumbai probably due to the greater influx of populations from tribal areas. HbD was more frequent in children in Delhi where the Punjabi population is higher.

HbS is more frequently observed in the tribal populations, HbE in the eastern region, HbD in Punjab, and βTT to varying degrees in almost all population groups.[6] However, with the migration of the population across the country often for purposes of employment, there is an influx of the tribal population into the surrounding cities or into the larger cities including population movements from East to West, North or South, or vice-versa. With the intermixing of populations, there is likely to be the presence of unexpected hemoglobins in the populations under investigation.

Using the overall carrier frequency of βTT of 4.05% found in this study, the birth incidence of thalassemia can be calculated using the Hardy-Weinberg equation for recessively inherited single gene disorders. With current estimates of the Indian population being approximately 1.2 billion and a birth rate of 23/1000, the estimate of homozygous births would be 11,316 per year. This gives a fairly accurate assessment of the homozygote thalassemic load. The observed incidence in different population groups and/or region will permit National Programs for Thalassemia to be planned and carried out with greater precision and assurance.

Although antenatal diagnosis is central to the control of thalassemia, screening programs form an important and integral part of the programs. Several Mediterranean and western countries have achieved a significant change in the homozygote population since the last two decades.[15] Other countries which also have Thalassemia Control Programs include Canada,[16] Israel,[17] Turkey,[18] Thailand,[19] Lebanon,[20] West Bank and Gaza Strip,[21] Malaysia,[22] China,[23] Iran,[24] Egypt,[25] and Pakistan.[14] In India, some screening and few antenatal programs[26–32] are also effective. However, several more centers are required. In these programs, the population was essentially screened with follow-up of awareness of their thalassemic status and the need for reduction in the birth of β-thalassemic homozygotes through ante-natal diagnosis.

Populations to be screened include adolescents of high school/college for assessment of the β-thalassemic status along with education and awareness of the disease, as well as pre- and post-marriage counseling. Screening of women early in pregnancy for βTT and of their spouses, if indicated, for antenatal diagnosis for reducing the birth of homozygotes is important. Extended family screening of thalassemics allows identification of large majority of population at risk by screening only 13% of the population.[1]

The importance of screening programs lies in the fact that they also provide a platform for increased awareness and education regarding thalassemia in the screened population and the associated population group including parents, teachers, friends, siblings, and employees. Screening may be voluntary or mandatory.

β-thalassaemia carriers in a family are now easily detected using well-calibrated automated hematology cell counters and automated dedicated HPLC systems. However, the prevention program including early screening of pregnant women and spouse of thalassemic pregnant women for antenatal diagnosis and termination of a homozygote fetus has been slow in India due to several factors. These include late reporting of pregnancy and the lack of widespread facilities for screening and antenatal diagnosis. Screening of extended family in large centers is improving;[33] however, a large number of the extended families do not appear to comprehend the problem and some prefer not to be investigated.

Debates on whether screening high school/college students along with thalassemia awareness programs will succeed in India have continued. The question also remains whether high school children screened and counseled would be sensitive to the information regarding thalassemia and whether they would remember their thalassemia status at the time of marriage. A 20-year-old study in high school children in Montreal, Canada,[16] suggests that this is an effective strategy. Screening programs for high school students are currently being used and recommended.[34] Premarital screening in the Indian population is still considered controversial. In Iran[35] and Turkey,[18] premarital screening of couples has been successful. This large program was started when termination of pregnancy was not considered an option. However, currently Iran has a thriving antenatal diagnostic program and births of homozygous thalassemics are considerably lower.[24] A highly successful campaign for the detection of beta-thalassaemia trait and prevention of the birth of β-thalassemia major babies in the isle of Menorca has resulted in the absence of the birth of even a single homozygote in the population.[36]

A multipronged approach including screening of high school/college students, premarital screening, and of the extended family of thalassemics along with antenatal diagnosis needs to be considered for this vast and ethnically diverse country. Education and awareness regarding thalassemics need to be accelerated urgently among medical practitioners, paramedics, the thalassemic and general population to reduce the morbidity and mortality and the financial and sociopsychological burden of the thalassemic families.

It has been estimated that the lifetime cost of healthcare, premature mortality, and lost earnings versus a national screening program including antenatal diagnosis in Israel[17] gives a cost–benefit ratio of 4.22:1 and adding a societal perspective 6.01:1. A recent report from Hong Kong[37] offers an almost similar cost–benefit ratio.

This study provides evidence that the birth rate of thalassemic homozygotes may be much higher than that stated in recent references.[4] Homozygote births are likely to be far higher in population groups with higher gene frequency. It is worthwhile carrying out screening programs, increasing awareness of the disease by education of the medical fraternity, the public, and particularly the β-thalassemia families to reduce the thalassemic homozygous population.
